# A Comparison of a Solid Organ Transplant Population‐Specific Antibiogram With the Hospital‐Wide Antibiogram

**DOI:** 10.1111/tid.70136

**Published:** 2025-11-25

**Authors:** Arzina Aziz Ali, Scott Borgetti, Alan E. Gross, Alfredo J. Mena Lora, Ryan Knodle, Taha Ali, Nahed Ismail

**Affiliations:** ^1^ University of Illinois at Chicago College of Medicine Chicago Illinois USA; ^2^ Northwest Suburban Medical Associates Arlington Heights Illinois USA

**Keywords:** antibiogram, antimicrobial resistance, empiric therapy, solid organ transplant

## Abstract

**Background:**

Solid organ transplant (SOT) recipients are vulnerable to infections with multidrug‐resistant organisms, and they often do not receive adequate empiric antimicrobials for serious Gram‐negative infections. Knowledge of differences in antimicrobial resistance rates in this specific population can help guide empiric antimicrobial choices.

**Methods:**

We performed a retrospective cohort study comparing antimicrobial susceptibility patterns of Gram‐negative organisms isolated from the adult SOT recipients with documented sepsis with the hospital‐wide inpatient antibiogram. To evaluate empiric antimicrobial coverage, we constructed a Weighted‐Incidence Syndromic Combination Antibiogram (WISCA) by calculating the proportion of susceptible isolates per antimicrobial agent. We then compared the coverage rates against our institution's sepsis treatment guidelines.

**Results:**

A total of 90 Gram‐negative isolates comprised the SOT cohort, while the inpatient antibiogram cohort had 1328 isolates. *Pseudomonas aeruginosa* (27%) and *Escherichia coli* (31%) were the most common isolates in the SOT and inpatient antibiogram cohorts, respectively. Overall, antimicrobial resistance rates were higher in the SOT population compared to the hospital‐wide inpatient antibiogram population. WISCA analysis showed imipenem was active for 59% of isolates as opposed to the institutional recommendation of piperacillin–tazobactam, which covered 39% of SOT isolates.

**Conclusion:**

Current empiric antimicrobial recommendations for SOT patients are based on data from the general patient population, which may not accurately reflect the resistance patterns in this unique population. Utilizing patient population‐specific antibiograms may improve empiric antimicrobial therapy and warrants further research.

## Graphical abstract

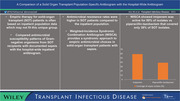

## 1 Introduction

Infections due to multidrug‐resistant organisms (MDROs) are a major cause of morbidity and mortality among solid organ transplant (SOT) recipients [1]. Therapeutic options for MDRO infections are limited and often more toxic, resulting in higher rates of mortality when compared with infections caused by susceptible organisms [2]. The World Health Organization (WHO) estimates that over 1.27 million deaths worldwide in 2019 were associated with MDROs [3]. In the United States, the CDC reported that between 2012 and 2017, MDRO pathogens caused over 2.8 million infections and 35 000 deaths annually [4]. SOT recipients are at an increased risk of MDRO infections due to their immunocompromised status, prolonged hospitalization, exposure to antibiotics, and presence of indwelling devices [5]. Studies show that the incidence of post‐transplant infections due to multidrug‐resistant Gram‐negative bacteria has increased [5]. Moreover, studies have also shown that SOT patients often do not receive adequate empiric antimicrobials for serious Gram‐negative infections [6, 7]. Therefore, understanding epidemiological differences in antimicrobial resistance rates in this specific population can help guide empiric antimicrobial choices for SOT recipients presenting with serious infections. The aim of our study was to compare differences in antimicrobial resistance of Gram‐negative bacteria isolated from SOT recipients with documented sepsis to resistance rates seen in the hospital‐wide antibiogram. Our study also explored the likelihood of effective empiric active antimicrobial coverage in the SOT cohort based on the use of antibiotics recommended in our institutional empiric sepsis treatment guidelines.

## 2 Methods

We conducted a retrospective cohort study of adult SOT recipients with documented sepsis, admitted to the University of Illinois Hospital between January 2021 and April 2023.

### 2.1 Study Setting and Population

The hospital is a 432‐bed academic tertiary care center located in Chicago, Illinois. According to the Organ Procurement and Transplantation Network, a total of 1091 SOT procedures were performed at our institution during the study period. These included kidney (*n* = 804), liver (*n* = 166), combined kidney‐pancreas (*n* = 104), intestinal (*n* = 10), and pancreas (*n* = 7) transplants. We included adult SOT recipients with at least one Gram‐negative bacterial isolate from any clinical site and documentation of sepsis in the electronic medical record.

### 2.2 Data Collection and Comparison

We compared antimicrobial susceptibility patterns of Gram‐negative organisms isolated from the SOT cohort with those in the hospital‐wide inpatient antibiogram. The hospital antibiogram included data from September 2021 to August 2022 and followed the Clinical and Laboratory Standards Institute (CLSI) guidelines (2020, 30th edition), incorporating only the first isolate per patient. Susceptibility percentages were calculated, and odds ratios (ORs) with 95% confidence intervals (CIs) were used for statistical comparisons. The study was approved by the hospital's institutional review board (IRB).

### 2.3 Empiric Therapy Evaluation via WISCA

To assess empiric antimicrobial coverage, we constructed a Weighted‐Incidence Syndromic Combination Antibiogram (WISCA) based on the SOT cohort. The WISCA method, originally described by Hebert et al., uses a syndrome‐specific approach to evaluate the probability that a given antimicrobial or combination regimen will be effective against all likely pathogens in a clinical scenario. For this analysis, we calculated the proportion of susceptible isolates per agent by dividing the number of susceptible isolates by the total number of Gram‐negative isolates in the SOT cohort. Coverage rates were compared against our institution's sepsis treatment guidelines.

## 3 Results

A total of 62 unique patients with 90 Gram‐negative isolates comprised the SOT cohort, while the inpatient antibiogram cohort had 1328 isolates. Among the 62 unique patients, 26 (41.9%) were kidney transplant recipients, 18 (29.0%) were liver transplant recipients, 10 (16.1%) had undergone kidney‐pancreas transplantation, 5 (8.1%) had a combined liver‐kidney transplant, and 3 (4.8%) had undergone small bowel transplantation. Among SOT cohort culture sources, the most common was blood, accounting for 38 out of 90 isolates (42.2%), followed by respiratory samples (BAL, tracheal cultures) with 22 isolates (24.4%), and urine with 14 isolates (15.6%). Less commonly, isolates were obtained from abdominal body fluid, pleural fluid, and tissue.

In the SOT group, *Pseudomonas aeruginosa* was the most common organism (24 out of 90 cases, 27%), while *Escherichia coli* was most common hospital‐wide (409 out of 1328 cases, 31%). *Pseudomonas* isolates in the SOT cohort (*n* = 24) were significantly less susceptible to common antipseudomonal beta‐lactams compared to the inpatient antibiogram cohort (*n* = 262), including piperacillin–tazobactam (11/24 [46%] vs. 189/262 [72%]; OR 0.33, 95% CI 0.13–0.83), imipenem (8/24 [33%] vs. 183/262 [70%]; OR 0.21, 95% CI 0.08–0.56), and cefepime (10/24 [42%] vs. 183/262 [70%]; OR 0.31, 95% CI 0.12–0.80). *E. coli* isolates in the SOT cohort (*n* = 16) were also significantly less susceptible to piperacillin–tazobactam (8/16 [50%] vs. 319/409 [78%]; OR 0.28, 95% CI 0.09–0.89) compared to the inpatient antibiogram (*n* = 409), but not significantly less likely to be susceptible to imipenem (15/16 [94%] vs. 409/409 [100%]; OR 0, 95% CI 0–1.53) or cefepime (12/16 [75%] vs. 344/409 [84%]; OR 0.57, 95% CI 0.17–2.50). Table 1 shows susceptibility differences between SOT and inpatient antibiogram cohorts for the most common organisms isolated and for commonly used beta‐lactam agents. WISCA analysis showed imipenem was active for 59% of isolates as opposed to the institutional recommendation of piperacillin–tazobactam, which covered 39% of SOT isolates (Table 2). Among other non‐B‐lactams, levofloxacin provided coverage for 51% of the isolates, and trimethoprim‐sulfamethoxazole covered 38%.

**TABLE 1:** Comparison of antimicrobial susceptibility in solid organ transplant versus hospital‐wide inpatient isolates.

SOT cohort susceptibility % vs. inpatient antibiogram cohort susceptibility %				
Species	No. of isolates (*N*) SOT vs. Inpatient antibiogram	Cefepime	Piperacillin–tazobactam	Imipenem
*Pseudomonas aeruginosa*	24 / 262	42% / 70%	46% / 72%	33% / 70%
*Escherichia coli*	16 / 409	75% / 84%	50% / 78%	94% / 100%
*Klebsiella pneumoniae*	10 / 198	30% / 71%	30% / 67%	90% / 92%
*Proteus mirabilis*	4 / 113	25% / 87%	0% / 87%	50% / ND
*Enterobacter cloacae*	8 / 81	75% / 75%	63% / 65%	88% / 96%

*Note*: Susceptibility rates are shown for the most commonly isolated organisms and for commonly used beta‐lactam antibiotics. ND: not enough data available to determine susceptibility rate; susceptibility not reported in at least one isolate.

**TABLE 2:** Weighted‐Incidence Syndromic Combination Antibiogram for SOT patients with Gram‐negative sepsis.

	Infections covered (%)
Antimicrobial regimen	Sepsis
Imipenem	59
Levofloxacin	51
Ceftazadime	50
Cefepime	50
Piperacillin–tazobactam	39
TMP‐SMX	38
Ceftriaxone	34

*Note*: A total of 90 bacterial isolates. For agents that are not reported for a given pathogen (such as pip‐tazo for *Serratia*, ceftriaxone for *Acinetobacter*, etc., due to FDA recommendations/limitations of the Vitek instrument), then the agent will be counted as nonsusceptible for the WISCA analysis.

## 4 Discussion

Antibiograms report antimicrobial susceptibility rates of local bacterial isolates and aid in selecting an empiric antimicrobial regimen. However, hospital‐wide inpatient antibiograms may not be reflective of susceptibility rates in specialized populations, such as SOT recipients with sepsis, due to their increased risk of infections with MDROs. These differences can impact the effectiveness of the empiric treatment and patient outcomes [8]. Therefore, our study evaluated the differences in susceptibility rates between the two populations to select an SOT‐specific empiric sepsis antimicrobial agent.

Our results demonstrate that overall antimicrobial resistance rates were higher in the SOT population compared to the hospital‐wide inpatient antibiogram population. This finding aligns with previous studies [8, 9, 10, 11]. Rosa et al. compared blood and urine isolates of *E. coli*, *Klebsiella pneumonia*, and *P. aeruginosa*—the most frequently isolated Gram‐negative isolates—and found SOT isolates had significantly lower susceptibility to first and second‐line antibiotics compared to the hospital‐wide antibiogram [8]. Similarly, in our study, *E. coli*, *K. pneumonia*, and *P. aeruginosa* were the most common Gram‐negative bacterial isolates in both SOT and hospital‐wide antibiograms. Koryam et al. made a UTI‐specific antibiogram in renal transplant recipients and compared it with the institutional antibiogram and found that *E. coli* isolates in this patient population were significantly more resistant to multiple antibiotic drug classes compared with the general hospital population [11]. Pilmis et al. reviewed studies on MDR Gram‐negative organisms in renal and liver transplant patients, reporting a high prevalence of MDR pathogens in these populations (15%–20% in liver transplant recipients and 14%–17% renal transplant recipients). In addition, they found a high prevalence of infections caused by MDR pathogens, occurring in 30%–65% of liver transplant recipients and 50%–60% of renal transplant recipients. Similar to our study, this high prevalence of MDR pathogens in transplant patients challenges the choice of empiric antimicrobial therapy in this special population [9].

Hebert et al. developed a novel antibiogram, called WISCA, designed to assess the likelihood that an antibiotic or combination of antibiotics will effectively treat all the relevant organisms in a patient with that syndrome, such as sepsis in our case. This contrasts with the traditional antibiogram, which does not provide syndrome‐specific antimicrobial guidance or account for the distribution of various pathogens associated with a particular syndrome [12]. In our study, WISCA for SOT patients with Gram‐negative sepsis showed that imipenem was a more active empiric agent compared to the institutional recommendation of piperacillin–tazobactam. However, imipenem covered only 59% of the isolates. As shown in Table 1, this reduced activity is primarily driven by *P*. aeruginosa, while other Gram‐negative organisms have largely retained susceptibility. Other potential reasons for this limited coverage include the presence of carbapenem‐resistant *P. aeruginosa*, intrinsic resistance in organisms such as *Stenotrophomonas*, and the lack of susceptibility testing for certain pathogens, such as *Acinetobacter baumannii* complex.

There are several limitations to our study. Our study is retrospective, and our SOT cohort has a small sample size of 90 Gram‐negative isolates; as such, it does not meet the CLSI recommended threshold of 30 isolates per organism for the SOT population. Despite this limitation, our findings highlight higher resistance rates among SOT patients, providing important insight into this high‐risk group. Furthermore, the use of WISCA also addresses this limitation by combining susceptibility data across relevant organisms, providing a syndromic approach to empiric antimicrobial choices in SOT patients with sepsis.

While the CLSI recommends including only the first isolate per patient per time period in the antibiogram, in our study, we included the first isolate per patient per hospitalization with a sepsis diagnosis, as the aim of our study was to focus on the SOT patients admitted with sepsis.

Current empiric antimicrobial recommendations and stewardship interventions for SOT patients are based on data from the general patient population. However, as our study and other prior studies have shown, it may not accurately reflect the unique pathogen burden, infection risks, and outcomes specific to SOT patients [10]. Further studies are needed to evaluate the effectiveness of utilizing patient population‐specific antibiograms and syndrome‐specific antimicrobial guidance, such as WISCA, in optimizing empiric antimicrobial therapy.

## Funding

The authors have nothing to report.

## Conflicts of Interest

Scott Borgetti: GSK (Grant/Research Support). Alan E. Gross: Becton Dickinson Co (Advisor/Consultant). The other authors declare no conflicts of interest.

## References

[1] A. Nellore and R. A. Lee . “Multidrug‐Resistant Organisms: Pre‐Transplant Evaluation and Management,” in Emerging Transplant Infections: Clinical Challenges and Implications, ed. M. I. Morris , C. N. Kotton , and C. R. Wolfe (Springer International Publishing, 2021), 287–312.

[2] A. Babiker , G. Karadkhele , A. Bombin , et al., “The Burden and Impact of Early Post‐Transplant Multidrug‐Resistant Organism Detection Among Renal Transplant Recipients, 2005–2021,” Open Forum Infectious Diseases 11, no. 3 (2024): ofae060, 10.1093/ofid/ofae060. 38464488 PMC10924447

[3] “Antimicrobial Resistance,” WHO , published November 21, 2023, https://www.who.int/news‐room/fact‐sheets/detail/antimicrobial‐resistance#:~:text=Key%20facts,income%20countries%20are%20most%20affected.

[4] P. D. Tamma , E. L. Heil , J. A. Justo , A. J. Mathers , M. J. Satlin , and R. A. Bonomo , “Infectious Diseases Society of America 2024 Guidance on the Treatment of Antimicrobial‐Resistant Gram‐Negative Infections,” Clinical Infectious Diseases ahead of print, August 07, 2024, 10.1093/cid/ciae403. 39108079

[5] S. M. Pouch and G. Patel , “Multidrug‐Resistant Gram‐Negative Bacterial Infections in Solid Organ Transplant Recipients—Guidelines From the American Society of Transplantation Infectious Diseases Community of Practice,” Clinical Transplantation 33, no. 9 (2019): e13594, 10.1111/ctr.13594. 31102483

[6] B. Hamandi , A. M. Holbrook , A. Humar , et al., “Delay of Adequate Empiric Antibiotic Therapy Is Associated With Increased Mortality Among Solid‐Organ Transplant Patients,” American Journal of Transplantation 9, no. 7 (2009): 1657–1665, 10.1111/j.1600-6143.2009.02664.x. 19459798

[7] M. Green , “Introduction: Infections in Solid Organ Transplantation,” American Journal of Transplantation 13 (2013): 3–8, 10.1111/ajt.12093. 23464993

[8] R. Rosa , J. Simkins , J. F. Camargo , O. Martinez , and L. M. Abbo , “Solid Organ Transplant Antibiograms: An Opportunity for Antimicrobial Stewardship,” Diagnostic Microbiology and Infectious Disease 86, no. 4 (2016): 460–463, 10.1016/j.diagmicrobio.2016.08.018. 27733304

[9] B. Pilmis , E. Weiss , A. Scemla , et al., “Multidrug‐Resistant Enterobacterales Infections in Abdominal Solid Organ Transplantation,” Clinical Microbiology and Infection 29, no. 1 (2023): 38–43, 10.1016/j.cmi.2022.06.005. 35716912

[10] J. Tessier , “A Work in Progress: Antimicrobial Stewardship in Solid Organ Transplant Patient Populations,” Current Opinion in Infectious Diseases 35, no. 4 (2022): 363–369, 10.1097/QCO.0000000000000848. 35849527

[11] G. B. Korayem , T. T. Zangeneh , and K. R. Matthias , “Recurrence of Urinary Tract Infections and Development of Urinary‐Specific Antibiogram for Kidney Transplant Recipients,” Journal of Global Antimicrobial Resistance 12 (2018): 119–123, 10.1016/j.jgar.2017.08.009. 28859935

[12] C. Hebert , J. Ridgway , B. Vekhter , E. C. Brown , S. G. Weber , and A. Robicsek , “Demonstration of the Weighted‐Incidence Syndromic Combination Antibiogram: An Empiric Prescribing Decision Aid,” Infection Control & Hospital Epidemiology 33, no. 4 (2012): 381–388, 10.1086/664768. 22418634

